# Postexercise urinary alpha-1 acid glycoprotein is not dependent on hypoxia

**DOI:** 10.1152/japplphysiol.00476.2021

**Published:** 2021-11-11

**Authors:** Kelsey E. Joyce, George M. Balanos, Christopher Bradley, Amy Fountain, Arthur R. Bradwell, Samuel J. E. Lucas

**Affiliations:** ^1^School of Sport, Exercise and Rehabilitation Sciences, University of Birmingham, Birmingham, United Kingdom; ^2^The Binding Site Ltd., Birmingham, United Kingdom; ^3^Medical School, University of Birmingham, Birmingham, United Kingdom

**Keywords:** alpha-1 acid glycoprotein, exercise, hypoxia, orosomucoid, proteinuria

## Abstract

Proteinuria is a transient physiological phenomenon that occurs with a range of physical activities and during ascent to altitude. Exercise intensity appears to dictate the magnitude of postexercise proteinuria; however, evidence also indicates the possible contributions from exercise-induced hypoxemia or reoxygenation. Using an environmental hypoxic chamber, this crossover-designed study aimed to evaluate urinary alpha-1 acid glycoprotein (α1-AGP) excretion pre/postexercise performed in hypoxia (HYP) and normoxia (NOR). Sixteen individuals underwent experimental sessions in normoxia (NOR, 20.9% O_2_) and hypoxia (HYP, 12.0% O_2_). Sessions began with a 2-h priming period before completing a graded maximal exercise test (GXT) on a cycle ergometer, which was followed by continuation of exposure for an additional 2 h. Physiological responses (i.e., blood pressure, heart rate, and peripheral oxygenation), Lake Louise Scores (LLSs), and urine specimens (analyzed for albumin and α1-AGP) were collected pre- and postexercise (after 30, 60, and 120 min). Peak power output was significantly reduced in HYP (193 ± 45 W) compared with NOR (249 ± 59 W, *P* < 0.01). Postexercise urinary α1-AGP was greater in NOR (20.04 ± 14.84 µg·min^−1^) than in HYP (15.08 ± 13.46 µg·min^−1^), albeit the difference was not significant (*P* > 0.05). Changes in urinary α1-AGP from pre- to post-30 min were not related to physiological responses or performance outcomes observed during GXT in NOR or HYP. Despite profound systemic hypoxemia with maximal exercise in hypoxia, postexercise α1-AGP excretion was not elevated above the levels observed following normoxic exercise.

**NEW & NOTEWORTHY** By superimposing hypoxic exposure and maximal exercise, we were able to investigate the impact of hypoxia on postexercise proteinuria. Urinalysis for α1-AGP (via particle-enhanced immunoturbidimetry) in specimens collected pre-/postexercise enabled the sensitive detection of altered glomerular permeability. Data indicated that exercise intensity, rather than the degree of exercise-induced hypoxemia, determines postexercise proteinuria.

## Graphical abstract

**Figure:**
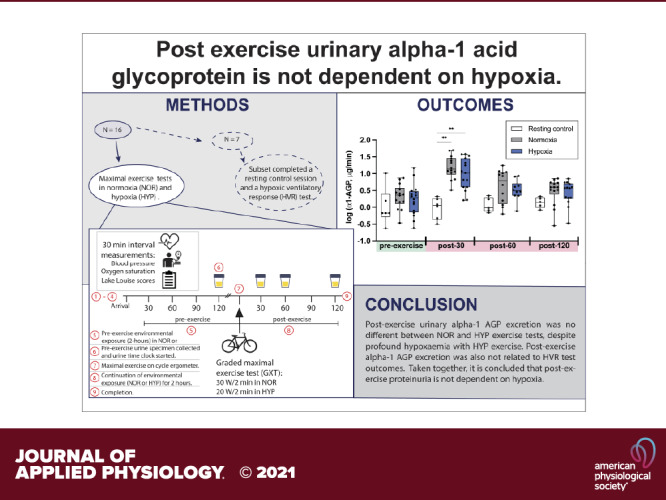

## INTRODUCTION

Glomerular proteinuria is a transient physiological phenomenon that occurs in healthy individuals following exercise (1) and during ascent to altitude (2, 3). Exhibited across a range of physical activities [e.g., swimming (4), running (5), and rowing (6)], it can be characterized by increases in urinary albumin or alpha-1 acid glycoprotein (α1-AGP; 1, 7), the latter being a potentially more sensitive marker of proteinuria (3). Exercise intensity ultimately dictates the degree of the postexercise increases (8, 9), although the mechanism(s) for this are not well understood. Intermittent exercise has been shown to elicit greater increases in proteinuria compared with continuous exercise (10), implicating a possible contribution from exercise-induced hypoxemia (or reoxygenation; 11). This is supported by findings from altitude studies that have shown relationships between urinary α1-AGP and blood oxygenation (12). Furthermore, hypoxia (HYP) is known to potentiate a variety of factors that have been implicated for postexercise proteinuria such as increased oxidative stress (13), acid-base balance disruption (8, 14), and increased blood pressure via changes in catecholamines (15) or elements of the renin-angiotensin-aldosterone-system (16, 17).

Exercising at altitude presents a unique way to investigate the involvement of hypoxemia in the development of postexercise glomerular proteinuria. Hypoxic ventilatory response (HVR) tests would support such investigation given that HVR relates to the degree of hypoxemia exhibited with exercise at altitude (18). Therefore, it was hypothesized that: *1*) exercise under hypoxic conditions would elicit greater postexercise α1-AGP excretion compared with a sea-level equivalent exercise bout and *2*) a greater HVR may attenuate exercise-induced hypoxemia and thereby limit postexercise elevations in proteinuria.

To evaluate the impact of hypoxemia on postexercise glomerular proteinuria, the objective of this study was to measure α1-AGP excretion following maximal exercise in an environmental chamber. This study aimed to examine: *1*) the time course and degree of urinary α1-AGP excretion surrounding exercise in normoxia (NOR) and hypoxia (HYP); *2*) physiological responses to exercise in NOR and HYP in relation to postexercise urinary α1-AGP excretion; and *3*) whether any associations existed between HVR test outcomes and the degree of postexercise urinary α1-AGP (exhibited in NOR vs. HYP).

## METHODS

### Participants and Design

Ethical approval was granted by the University of Birmingham (ERN_18-1270). Written informed consent was obtained before participation. Consented participants completed a general health history questionnaire to screen for signs, symptoms, or history of cardiovascular, pulmonary, renal, or metabolic disease with only healthy individuals included. Individuals who reported any of the following were also excluded: *1*) active pharmacotherapy, *2*) actively being under the care of a GP (for any reason), or *3*) history of smoking. If a participant exhibited any of the following (after repeated measurements) at rest during any session, they were withdrawn: *1*) systolic blood pressure (SBP) ≥ 140 mmHg, *2*) diastolic blood pressure (DBP) ≥ 90 mmHg, or *3*) heart rate (HR) > 100 beats·min^−1^.

All participants first completed a familiarization session that included a graded incremental maximal exercise test (GXT) in ambient conditions (see *Graded Incremental Maximal Exercise Tests*). A randomized crossover design was then adopted with all participants completing two experimental sessions (NOR and HYP; see *Experimental Sessions*), with each consisting of physiological response measurements, a GXT, and timed urine collections (see *Urine Experiments*). Resting control trials and HVR tests (see *Hypoxic Ventilatory Response Tests*) were also completed in a subset of participants. For all sessions, participants were asked to refrain from *1*) partaking in strenuous exercise or consuming alcoholic beverages 24 h prior and *2*) consuming caffeine 4 h prior. All sessions were separated by no less than 48 h.

### Experimental Sessions

**Figure 1.**
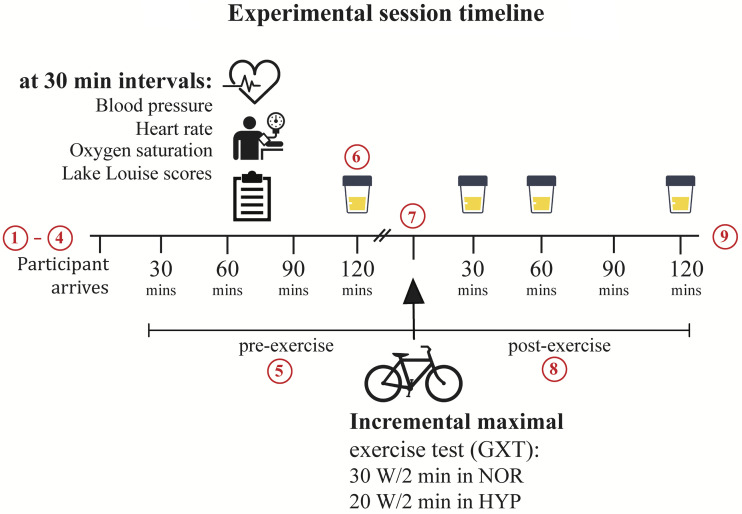
Experimental session timeline. Detailed schematic for the conduct of resting control, normoxic (NOR), and hypoxic (HYP) sessions. Resting control sessions were identical to NOR and HYP sessions other than the graded incremental maximal exercise (GXT), which was substituted with a resting period (length of familiarization GXT). Each session proceeded as follows: *1*) participant arrived and underwent 10–15 min of seated rest; *2*) “baseline” (sea-level) physiological responses (heart rate, systolic and diastolic blood pressures, and peripheral oxygenation) and Lake Louise Scores were recorded; *3*) participant emptied their bladder, which started the clock for timed urine collections; *4*) participant entered the chamber; *5*) participant was encouraged to drink at least 500 mL of water in the 2 h leading up to GXT during which physiological response outcomes were recorded at 30-min intervals; *6*) urine specimens collected immediately preexercise; *7*) GXT was administered with staged increases adjusted for condition (30 W vs. 20 W/2 min; NOR and HYP, respectively; to standardize test duration between conditions) and physiological response outcomes recorded throughout (every 1–2 min; refer to *Graded Incremental Maximal Exercise Tests*); *8*) postexercise physiological response outcomes and timed urine specimens collected at 30, 60, and 120 min following GXT; and *9*) participant exited the environmental chamber and was monitored for approximately 15 min (and up to 1 h) before departure.

Experimental sessions in NOR (20.9% O_2_) and HYP (∼12.0% O_2_, equivalent to 4,000 m) were conducted in an environmental chamber (TIS Services, Hampshire, UK) as outlined in Fig. 1. Participants were blinded to condition wherever possible [e.g., using sham hypoxia for NOR sessions (19)]. Sessions consisted of a preexercise 2-h priming exposure (upright seated), during which participants were encouraged to consume 500 mL of water, before completing a GXT that lasted ∼15 min. The GXT was followed by a continuation of exposure for an additional 2 h (total session duration, 4.5 h). Resting control sessions were identical to experimental sessions except GXT, which was substituted with a time-controlled resting period (familiarization GXT duration used). If at any point a participant felt too unwell to continue, presented with moderate or severe symptoms of acute mountain sickness [i.e., Lake Louise Score, LLS, >5 (20)], or requested to leave the chamber for any reason, they were removed immediately.

Peripheral oxygenation ($Sp_{O_{2}}$; via finger pulse oximetry; WristOx_2_, Model 3150, Nonin Medical Inc., Plymouth, MN), heart rate (HR; via 3-lead electrocardiogram, ECG), SBP and DBP (via automated sphygmomanometry of the brachial artery; Tango M2 Stress Monitor, SunTech Medical, Morrisville, NC), and LLS were measured: upon arrival, at 30-min intervals throughout experimental sessions, and for at least 15 min (and up to 1 h) following each session. Mean arterial pressure (MAP, mmHg) at 30-min intervals was calculated from resting SBP, DBP, and HR using a weighted formula (21). Urine specimens were also collected throughout these sessions as outlined in *Specimen collection and handling*.

### Graded Incremental Maximal Exercise Tests

Graded exercise tests were conducted on an upright cycle ergometer (Velotron, Quarq Technology, Spearfish, SD) and began with a 3-min warm-up, which was performed at 50 W and had a target rating of perceived exertion (RPE) of 14 [Borg Scale (22)]. Following the warm-up, a 2-min stage was performed at 60 W. To control for total exercise duration between conditions, wattage was increased every 2 min by 20 W or 30 W (in HYP and NOR, respectively) until volitional fatigue (23) or an RPE of 20 was achieved. To ensure maximal effort in both, NOR and HYP, the anticipated maximal power output (W_max_) for each condition was estimated from familiarization W_max_ (i.e., NOR = 100% of familiarization W_max_ and HYP = 70%–80% of familiarization W_max_). After W_max_ was achieved an unloaded, self-paced cool-down was performed for 3–5 min.

$Sp_{O_{2}}$ and HR were recorded each minute throughout GXT, whereas power output (in watts), SBP, DBP, and RPE were recorded at the end of each 2-min stage. Performance outcomes (maximal power output, W_max_) and physiological response outcomes (SBP_max_, DBP_max_, HR_max_, and $Sp_{O_{2max}}$) were also recorded at the time maximal effort was achieved. MAP_max_ was calculated using the previously mentioned weighted formula by inputting SBP_max_, DBP_max_, and HR_max_ measurements. Blood lactate (BLa) was analyzed (Lactate Plus, Nova Biomedical, Waltham, MA) before and after GXT from blood obtained by finger prick. Delta BLa (Δ BLa) was estimated from these measurements.

### Urine Experiments

#### Specimen collection and handling.

To mark the start of timed urine collections, participants emptied their bladder (not collected) within 15–20 min of arrival and before entering the environmental chamber. All urine produced thereafter was collected into 3,000 mL, UV-protected, polyethylene containers designed for human urine collection (SARSTEDT, Nümbrecht, Germany). Separate containers were used for each timed collection with specimens collected immediately preexercise (arrival to preexercise; ∼2 h) and at the following timepoints following exercise: post-30 min (preexercise to post-30 min; GXT duration +30 min), post-60 min (post-30 to post-60 min; 30 min), and post-120 min (post-60 to post-120 min; 60 min; see Fig. 1). Specimen volume (mL), weight (g), and collection time (h:min) were immediately recorded upon collection with a sample (20 mL), then aliquoted into a conical centrifuge tube and temporarily stored on ice (0°C) until the end of the session, when all samples were centrifuged (5,400 rpm at 21°C) for 10 min. Following centrifugation, supernatants were further aliquoted into 2 mL cryovials (4 × replicates) and frozen at –80°C until urinalysis.

#### Urinalysis.

Urine specimens were thawed in a warming cabinet (37°C) for 1 h before urinalysis. Particle-enhanced immunoturbidimetry for low-concentration α1-AGP (measuring range: 0.08–148.20 mg·L^−1^) and albumin (measuring range: 11–66,500 mg·L^−1^) was performed using the Optilite autoanalyzer (The Binding Site, Ltd., Birmingham, UK). Triplicate (α1-AGP) results were used in conjunction with specimen volume (or weight) and collection duration to estimate α1-AGP excretion rate (µg·min^−1^). Triplicate excretion rates for each specimen were then averaged with this result used for statistical analysis. Change (Δ) in α1-AGP was estimated as the difference between preexercise and post-30-min excretion.

### Hypoxic Ventilatory Response Tests

An additional visit was undertaken among a subset of participants (*n* = 6) to assess their HVR. HVR tests were administered via a dynamic end-tidal forcing (DEF) system (v1, BreathDP, University of Oxford) using a stepped, isocapnic-hypoxia protocol (24). Tests began with participants seated at rest while breathing ambient room air for 5 min via a mouthpiece connected to the DEF, which allowed ventilation to stabilize. During this resting period, “baseline” partial pressure of end-tidal CO_2_ ($\text{P}\text{E}{\text{T}}_{\text{C}{\text{O}}_{2}}$) was recorded. Next, a series of 3-min stepped decreases in the partial pressure of end-tidal oxygen ($\text{P}\text{E}{\text{T}}_{{\text{O}}_{2}}$) were administered (at 100 mmHg, 65 mmHg, and 55 mmHg) with $\text{P}\text{E}{\text{T}}_{\text{C}{\text{O}}_{2}}$ clamped ∼1 mmHg above “baseline” throughout. Three-minute steps were followed by a final 5-min step at 100 mmHg $\text{P}\text{E}{\text{T}}_{{\text{O}}_{2}}$ with total test duration being ∼20 min. Measures of ventilation (tidal volume; respiratory rate; and minute ventilation, V̇e), $Sp_{O_{2}}$, and HR (via 3-lead ECG) were recorded continuously throughout.

Thirty-second averages were estimated for V̇e, $Sp_{O_{2}}$, and V̇e/$Sp_{O_{2}}$ (L·min^−1^·%^−1^), and plotted as a function of time for the three isocapnic-hypoxic steps. V̇e/$Sp_{O_{2}}$ represented the direct relationship between V̇e and $Sp_{O_{2}}$ at each 30-s interval, whereas V̇e/$Sp_{O_{2}}$ slope (or ΔV̇e/Δ$Sp_{O_{2}}$) represented the slope of the best-fit line plotted (within individuals) from the linear regression (V̇e, *y*-axis; and $Sp_{O_{2}}$, *x*-axis) across all steps (25–27). Peak V̇e (L·min^−1^), minimum $Sp_{O_{2}}$ (%), and mean values for V̇e, $Sp_{O_{2}}$, and V̇e/$Sp_{O_{2}}$ were reported for each step, whereas V̇e/$Sp_{O_{2}}$ slope (ΔV̇e/Δ$Sp_{O_{2}}$) was reported for the entire HVR test (accounting for all three steps). By convention, V̇e/$Sp_{O_{2}}$ slopes were presented as absolute values with actual values used for statistical analysis. Void of missing data for all steps, repeated-measures ANOVA with Tukey’s post hoc test was used to compare HVR outcomes between isocpanic-hypoxic steps.

### Statistical Analysis

Normality of distribution was assessed using Shapiro–Wilk test for each outcome measure with data log-transformed where possible and outliers removed (using ROUT method) where appropriate (for normally distributed data) before analysis. For reference, raw α1-AGP excretion data were reported, however, analyses for urinary α1-AGP were conducted using log-transformed values as previously described (7).

Mixed-effects analysis was performed to evaluate the main effects of condition and time with *1*) Dunnett’s test performed when performing multiple comparisons against the resting control session (for urinary proteins only), *2*) Tukey’s correction for comparisons between conditions (i.e., rest vs. NOR vs. HYP) at multiple 30-min intervals, and *3*) Šidák’s correction for multiple comparisons between NOR and HYP (independent of resting control sessions) at 30-min intervals (for physiological responses and urinary proteins). Paired *t* test or Wilcoxon matched-pairs test were used to compare GXT outcomes (e.g., W_max,_ SBP_max_, DBP_max_, HR_max_, $Sp_{O_{2max}}$, and Δα1-AGP) between conditions. Statistical analysis for HVR test outcomes is outlined in *Hypoxic Ventilatory Response Tests*.

Correlation analyses (Pearson *r* or Spearman *rho*, where appropriate) were performed for log-transformed values of α1-AGP excretion [i.e., log(α1-AGP_post-30_) and log(α1-AGP_Δ_)] with a priori comparisons performed between GXT outcomes and HVR test outcomes (e.g., peak V̇e or ΔV̇e/Δ$Sp_{O_{2}}$). Linear regression analysis was also performed for any significant correlations with *R*^2^ presented for these relationships.

Statistical tests were performed using SPSS Statistics (v25 for Mac iOS, IBM, Armonk, NY) or Prism (v8.3.0 for Mac iOS, Graphpad Software Inc., San Diego, CA) with data presented as means ± SD unless otherwise indicated. All statistical tests were two tailed with significance set to α < 0.05.

## RESULTS

Eighteen individuals were enrolled with 16 (*n* = 16; 8 males, 8 females) university-aged (21.1 ± 1.3 yr) participants successfully completing both exercise experimental sessions. Attrition was attributed to one dropout (for personal reasons) and one researcher-initiated withdrawal (due to hypertension at rest in HYP) with any data collected from withdrawn participants excluded from the analysis. A subset of seven individuals also completed resting control trials, with six of these individuals also completing the HVR test.

### Experimental Sessions

**Figure 2.**
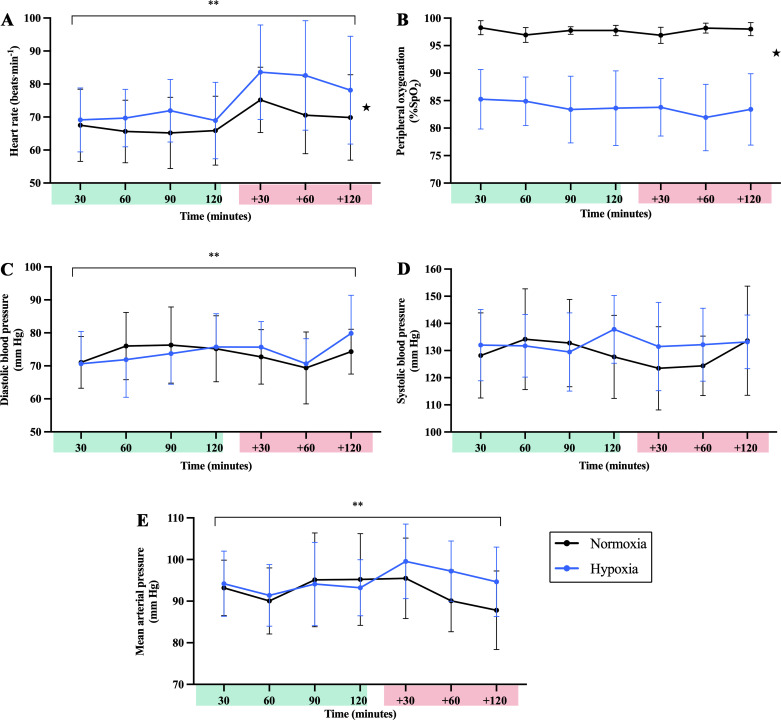
Physiological responses during normoxic (NOR) vs. hypoxic (HYP) sessions. *A*: heart rate (HR). *B*: peripheral oxygenation ($Sp_{O_{2}}$). *C*: diastolic blood pressure (DBP). *D*: systolic blood pressure (SBP). *E*: mean arterial pressure (MAP). Data are presented as means ± SD for 30-min interval measurements performed preexercise (green) and postexercise (red). *Significant main effect of condition observed for $Sp_{O_{2}}$ [*F*(1.00, 15.00) = 161, *P* < 0.001] and HR [*F*(1.00, 15.00) = 10.46, *P* = 0.006]. **Significant main effect of time observed for HR [*F*(2.44, 36.63) = 10.80, *P* < 0.001], DBP [*F*(3.82, 57.25) = 3.36, *P* = 0.017], and MAP [*F*(4.21, 63.16) = 2.81, *P* = 0.031].

Physiological responses from NOR and HYP sessions are presented in Fig. 2 with measurements from resting control trials (for the subset only) outlined in Supplemental Fig. S1 (see https://doi.org/10.6084/m9.figshare.14870136). Despite the significant effect of condition for $Sp_{O_{2}}$ (*P* < 0.001; Fig. 2*A*) and HR (*P* = 0.006, Fig. 2*B*), HYP sessions were well tolerated with LLS at 30-min intervals mostly being low (i.e., LLS < 3; mean of all measurements, 0.714 ± 0.781) or mild (i.e., LLS: 3–5 points) apart from one moderate score (i.e., LLS: 6–9 points) reported at the end of an HYP session (i.e., at post-120 min). No significant main effect of condition was observed for DBP (*P* = 0.771, Fig. 2*C*), SBP (*P* = 0.112, Fig. 2*D*), or MAP (*P* = 0.059, Fig. 2*E*).

A significant main effect of time was observed for HR (*P* < 0.001), DBP (*P* = 0.017), and MAP (*P* = 0.031) with these results likely attributable to similarities in the physiological responses following exercise (i.e., change from +30 to +120) in NOR and HYP. No significant main effect of time was observed for $Sp_{O_{2}}$ (*P* = 0.368) or SBP (*P* = 0.411). Similarly, no interaction effect between time and condition was observed for $Sp_{O_{2}}$ (*P* = 0.161), HR (*P* = 0.054), DBP (*P* = 0.242), SBP (*P* = 0.083), or MAP (*P* = 0.127).

### Maximal Exercise Tests

Results from paired *t* tests (or Wilcoxon tests) for GXT performance and physiological response outcomes are presented in Table 1. As expected, $Sp_{O_{2max}}$ and W_max_ were significantly lower in HYP than in NOR (both, *P* < 0.001), whereas SBP_max_ and MAP_max_ were significantly greater in HYP than in NOR (both, *P* < 0.05). DBP_max_, HR_max_, RPE, Δ BLa, and ambient CO_2_ were no different between NOR and HYP.

**Table 1.** Performance and physiological response outcomes from maximal graded exercise tests

	Normoxia	Hypoxia			
	Means ± SD		Mean Differences (95% CI)	*t* Statistic (df)or *z*-score	*P* Value
W_max_, W	249 ± 59	193 ± 45	–57 (–69, –45)	10.01 (15)	<0.001**
SBP_max_, mmHg	175 ± 36	198 ± 25	23 (1, 45)	2.27 (15)	0.039**
DBP_max_, mmHg	78 ± 12	80 ± 16	2 (–7, 12)	0.53 (15)	0.605
MAP_max_, mmHg	126 ± 19	139 ± 17	13 (1, 24)	2.36 (15)	0.032**
HR_max_, beats·min^−1^*	186 ± 19	186 ± 20	–1 (–7, 11)	–0.085	0.932
$Sp_{O_{2max}}$, %*	95 ± 3	81 ± 7	–15 (–21, –10)	–3.520	<0.001**
RPE*	19 ± 2	19 ± 2	0 (–1, 1)	–0.250	0.803
ΔBLa, mmol·L^–1^*	9.6 ± 12.3	7.3 ± 7.3	2.4 (–14.7, 8.1)	–0.507	0.688
CO_2_, ppm*	855 ± 386	674 ± 301	–169 (–455, 1113)	–0.365	0.715

GXT outcomes are presented as means ± SD or median ± interquartile range (IQR), where marked by asterisk, *paired samples *t* tests (and *t* statistics) were used to compare between conditions (normoxia vs. hypoxia) for normally distributed data, whereas Wilcoxon matched-pairs tests (and *z*-scores) were used when distribution was abnormal (outcomes indicated by *). Similarly, mean differences (95% confidence intervals, CIs) were presented for normally distributed data and median differences (98% CIs) for data with an abnormal distribution. **Significant difference between normoxia vs. hypoxia. Subscript (_max_) refers to measurements obtained at the time maximal effort was achieved. BLa, blood lactate; CO_2_, ambient carbon dioxide; DBP, diastolic blood pressure; GXT, graded maximal exercise test; HR, heart rate; MAP, mean arterial pressure; RPE, rating of perceived exertion; SBP, systolic blood pressure; $Sp_{O_{2}}$, peripheral oxygenation; W_max_, maximal power output.

### Urinalysis

**Figure 3.**
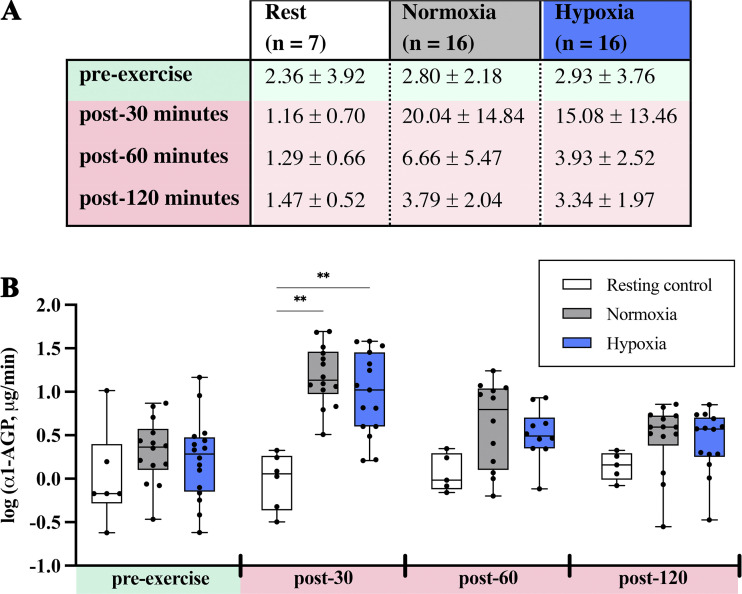
Urinary alpha-1 acid glycoprotein (α1-AGP) excretion rates. *A*: α1-AGP excretion (means ± SD, µg·min^−1^) during resting control sessions (among a subset of participants, *n* = 7) and surrounding exercise tests performed in normoxia and hypoxia (among the entire cohort, *n* = 16). *B*: corresponding box and whisker plots for log-transformed α1-AGP excretion rates (median ± 25% and 75% quartiles range; whiskers: 5th–95th percentiles). A mixed-effects analysis with Tukey’s test was used to compare log(α1-AGP) values between sessions (resting control vs. normoxia vs. hypoxia) at the 30-min intervals surrounding maximal exercise tests. Compared with resting control, log(α1-AGP) was significantly greater 30 min following exercise in both normoxia (*P* = 0.002) and hypoxia (*P* = 0.004). **All statistical tests were two-tailed with significance set to α < 0.05.

A grand total of 136 urine specimens were successfully collected from the 156 possible time points for NOR, HYP, and control sessions. In these specimens, urinalysis for α1-AGP was 95.6% successful, whereas urinalysis for albumin was only 23.7%. Urinary α1-AGP excretion rates (means ± SD, µg·min^−1^) from resting control, NOR, and HYP sessions are presented in Fig. 3 along with the corresponding plots and comparisons of log-transformed values.

Mixed-effects analysis comparing log(α1-AGP) values from resting control, NOR, and HYP sessions demonstrated the impact of exercise, as evidenced in the significant differences observed at post-30 min (as shown in Fig. 3*B*), as well as the significant main effects of condition [*F*(2.09, 31.34) = 10.82, *P* < 0.001] and time [*F*(1.40, 20.94) = 24.83, *P* < 0.001]. However, no interaction effect was apparent [*F*(2.15, 10.40) = 3.35, *P* = 0.073]. When log(α1-AGP) values were compared between NOR and HYP sessions independent of the resting control, no significant main effects were observed for condition [*F*(1.00, 15.00) = 3.88, *P* = 0.068] or interaction [*F*(2.50, 21.38) = 0.733, *P* = 0.519], however, the significant main effect of time remained apparent [*F*(2.72, 40.76) = 20.89, *P* < 0.001]. Consistent with the former, Δα1-AGP was no different between NOR (15.09 ± 14.77 µg·min^−1^) and HYP (11.22 ± 14.71 µg·min^−1^, *P* = 0.233).

### HVR Tests

**Figure 4.**
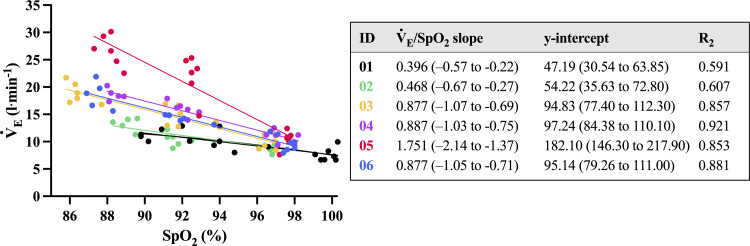
Linear regression analysis for minute ventilation (V̇e) and peripheral oxygenation ($Sp_{O_{2}}$). Thirty-second averages for V̇e (*y*-axis) and $Sp_{O_{2}}$ (*x*-axis) from hypoxic ventilatory response (HVR) tests are plotted for all isocapnic-hypoxic steps (partial pressures of end-tidal oxygen: 100 mmHg, 65 mmHg, and 55 mmHg). Linear regression analysis was applied to each participant’s (*n* = 6) individual HVR test data (18 data points per participant) with individual datasets plotted (in the figure) and represented (in the legend, ‘ID’ column) using a different color. Results from linear regression (i.e., slopes, *y*-intercepts, and R_2_ values of the best-fit lines) representative of V̇e/$Sp_{O_{2}}$ slope (or ΔV̇e/Δ$Sp_{O_{2}}$). By convention, V̇e/$Sp_{O_{2}}$ slopes are presented in the legend as absolute values (positive integers), although *y*-intercepts and 95% confidence intervals (CIs) are presented as actual values.

Mean and individual data for 30-s averages of V̇e, $Sp_{O_{2}}$, and V̇e/$Sp_{O_{2}}$ are presented in Supplemental Fig. S2 (see https://doi.org/10.6084/m9.figshare.14870154). As expected, a significant effect of $\text{P}\text{E}{\text{T}}_{{\text{O}}_{2}}$ on peak V̇e (*P* = 0.007) and mean V̇e (*P* = 0.006) was observed, with both increasing as $\text{P}\text{E}{\text{T}}_{{\text{O}}_{2}}$ was decreased. Similarly, $Sp_{O_{2}}$ was lower at each step, reflected in the significant main effect of $\text{P}\text{E}{\text{T}}_{{\text{O}}_{2}}$ on minimum and mean $Sp_{O_{2}}$ (both *P* < 0.001). Direct estimations of V̇e/$Sp_{O_{2}}$ (L·min^−1^·%^−1^) for each step also demonstrated a significant effect of $\text{P}\text{E}{\text{T}}_{{\text{O}}_{2}}$ on V̇e/$Sp_{O_{2}}$ (*P* = 0.008). By contrast, V̇e/$Sp_{O_{2}}$ slope (or ΔV̇e/Δ$Sp_{O_{2}}$), which was representative of the dynamic relationship between V̇e (*y*-axis) and $Sp_{O_{2}}$ (*x*-axis) for HVR tests, was plotted from data from all steps, albeit with linear regressions applied to individual 30-s interval data (as shown in Fig. 4).

#### Correlation analysis.

Results from correlation analyses are presented in Table 2. Significant positive relationships were observed between log(Δ α1-AGP) and SBP_max_ (*P* = 0.045; *R*^2^ = 0.317) and MAP_max_ (*P* = 0.040; *R*^2^ = 0.331) in HYP only. No other performance, physiological response, or HVR test outcomes were related to α1-AGP for either condition (Table 2). Of note, however, was the participant exhibiting the uncharacteristically elevated HVR (“ID 05” shown in red in Fig. 4). This participant was the only participant to exhibit greater post-30-min α1-AGP excretion in HYP (absolute, 33.70 µg·min^−1^; Δ 31.56 µg·min^−1^) than in NOR (absolute, 27.74 µg·min^−1^; Δ 26.26 µg·min^−1^), despite a reduction in W_max_ (33% reduction) that was greater than that of the group (23 ± 7% reduction). This participant also exhibited the second highest MAP_max_ (163 mmHg) out of the group in HYP.

**Table 2.** Correlation results for postexercise alpha-1 acid glycoprotein excretion

	Normoxia		Hypoxia	
	Log(α1-AGP_post-30_)	Log(α1-AGP_Δ_)	Log(α1-AGP_post-30_)	Log(α1-AGP_Δ_)
Exercise Tests	*P* Value	*P* Value	*P* Value	*P* Value
W_max_	0.347	0.450	0.234	0.129
$Sp_{O_{2max}}$	0.520†	0.508†	0.184†	0.398†
HR_max_	0.816†	0.573†	0.892†	0.867†
SBP_max_	0.674	0.726	0.072	0.045**
DBP_max_	0.458	0.477	0.554	0.501
MAP_max_	0.816	0.815	0.070	0.040**
HVR tests				
Peak V̇e	0.564†	0.497†	0.714†	0.714†
Minimum $Sp_{O_{2}}$	0.803†	0.919†	0.497†	0.497†
V̇e/$Sp_{O_{2}}$ slope	0.497†	0.419†	0.564†	0.564†

Correlation analyses were performed between log-transformed α1-AGP excretion rates [i.e., post-30 min or change (Δ) from pre- to post-30 min] and performance and physiological response outcomes from exercise tests, as well as hypoxic ventilatory responses (HVR) test outcomes using Pearson *r* (or Spearman *rho*, †). Linear regression was applied to data demonstrating a significant relationship. Significant relationships were evident between log(Δ) and SBP_max_ (*r* = 0.563, *R*^2^ = 0.317), as well as MAP (*r* = 0.564; *R*^2^ = 0.319) for hypoxia only. All statistical tests were two-tailed with significance set to α < 0.05. Significant results are indicated by double asterisk, **. V̇e/$Sp_{O_{2}}$ slope (or ΔV̇e/Δ$Sp_{O_{2}}$) represented the slope of the best-fit line plotted for the simple linear regression of data from all three isocapnic-hypoxic steps. α1-AGP, alpha-1 acid glycoprotein; DBP, diastolic blood pressure; HR_max_, maximal heart rate; MAP, mean arterial pressure; SBP, systolic blood pressure; $Sp_{O_{2}}$, peripheral oxygenation; V̇e, minute ventilation; W_max_, maximal power output.

## DISCUSSION

The present study assessed the contribution(s) of hypoxia to postexercise proteinuria by evaluating urinary α1-AGP excretion surrounding maximal exercise tests in NOR and HYP, as well as any association between HVR and the degree of postexercise α1-AGP excretion. In contrast to our original hypothesis, despite potent stimulation of hypoxemia with HYP exercise, marked reductions in $Sp_{O_{2}}$ were not accompanied by concomitant increases in postexercise α1-AGP excretion (refer to Fig. 3). In line with this, no significant relationships were observed between postexercise α1-AGP excretion and the degree of desaturation during exercise or HVR test outcomes (refer to Table 2). Nevertheless, findings from HVR tests were interesting with three types of responders reflected (as highlighted in Supplemental Fig. S2). Taken together, this evidence indicates that postexercise urinary α1-AGP excretion cannot be directly attributed to hypoxia.

The coupling between desaturation and attenuated postexercise α1-AGP excretion in HYP observed in this study supports earlier cycling-based studies that have demonstrated similar reductions (7) or unchanged postexercise excretion in hypoxia (28). The present observations were attributed to concomitant reductions in exercise performance known to accompany hypoxic exercise (29), which have, in part, been attributed to reductions in blood flow to the working limbs (30). It is suggested that in response to vigorous hypoxia (i.e., maximal exercise in HYP), an alteration in the redistribution of blood flow occurs, which promotes a balance between glomerular blood flow and pressure (31) that does not favor proteinuria to the same extent as NOR exercise [or lower exercise intensities (32)]. This implicates the likely role of renal hemodynamics in the progression of postexercise glomerular proteinuria (33). Development of significant relationships between postexercise α1-AGP and SBP_max_, as well as MAP_max_ in HYP, and despite attenuated α1-AGP excretion in the face of higher pressures (SBP_max_ and MAP_max_), further supports this and highlights the appropriation of intrinsic control.

Similarly, maladaptive renal hemodynamics (or related physiological factors) can provide an explanation for the increase in postexercise α1-AGP in HYP (compared with NOR) that occurred in the participant with the highest HVR (and greatest LLS), despite a reduction in power output. It was assumed that an augmented HVR would minimize postexercise proteinuria in HYP by limiting the degree of hypoxemia (18) or increasing exercise performance limitations (34); however, in HYP, a high HVR may perpetuate postexercise proteinuria by heightening the competition for blood flow between exercising muscles, ventilatory muscles, and the kidneys (35), and ultimately resulting in greater diffusion into Bowman’s space (36). Further investigation is required to evaluate the impact of HVR on proteinuria in this way. Similarly, further investigation of this anomaly will be important for understanding physiological excretion levels postexercise. Further, this observation supports the possibility that postexercise α1-AGP excretion, like albumin, may indicate underlying pathophysiology with, but not limited to, glomerular involvement (e.g., diabetes; 37). Taken together with α1-AGP urinalysis being more sensitive than albumin urinalysis (refer to *Urinalysis*), it is reasonable to conclude that α1-AGP urinalyses will afford identification of subclinical changes in glomerular responses, translating to earlier disease detection or improved status monitoring.

### Limitations and Future Directions

The absence of a prospective a priori power analysis for postexercise α1-AGP excretion may be considered a limitation of this study. Unfortunately, pre-/postexercise α1-AGP data were not available from the literature, making the effect size required to detect a difference between NOR and HYP unclear, which in turn prevented sample size the estimation. That said, biological relevance does not always coincide with indexed effect sizes (38). Thus, a contextualized approach, able to weigh various aspects of the experiment against the importance of reducing error, was considered to be most appropriate (39, 40). Nevertheless, results from this study should be interpreted in the context of the cohort studied.

Inability to report performance outcomes (e.g., exercise intensity) in relative measures (e.g., W·kg^−1^ or V̇o_2max_ in mL·kg^−1^·min^−1^) is also an obvious limitation and likely the cause for the absence of any significant relationship between power output and postexercise α1-AGP. Nevertheless, the purpose of this study was not to evaluate the effect of intensity but rather the effect of hypoxemia by delivering a potent hypoxic stimulus (i.e., maximal exercise in HYP). Moreover, the proteinuric effect of such hypoxic exercise was expected to transcend that of intensity.

Arguments against the expression of ventilatory responsiveness as a function of $Sp_{O_{2}}$ can be made. However, in this instance, such expression adequately shows the change in ventilation and also demonstrates its effectiveness at increasing $Sp_{O_{2}}$, notwithstanding the limitations of $Sp_{O_{2}}$ measurements. Further, although it is acknowledged that the relationship between Po_2_ and ventilation is hyperbolic (41), interpretation of ventilatory responsiveness using linear regression (applied to ventilation and $Sp_{O_{2}}$) was considered more appropriate in the context of the three isocapnic-hypoxic steps, which are representative of only a fragment of the hyperbolic response curve.

Building upon this study, and to improve understanding related to the role of renal hemodynamics in postexercise proteinuria, future researchers may consider: *1*) evaluating a larger cohort; *2*) analyzing physiological markers of sympathetic nerve activation (e.g., plasma epinephrine or norepinephrine), glomerular endothelial permeability (e.g., urinary glycosaminoglycans), or renal oxidative stress; and *3*) measuring renal blood flow (via nonradioactive microsphere) or perfusion (via near-infrared spectroscopy, NIRS).

### Conclusions

Findings demonstrate the utility of immunoturbidimetric analysis for urinary α1-AGP and examinations of postexercise proteinuria. Despite profound systemic hypoxemia, exercise-induced increases in urinary α1-AGP excretion appear to be dictated by exercise intensity.

## SUPPLEMENTAL DATA

10.6084/m9.figshare.14870136 Supplemental Fig. S1: https://doi.org/10.6084/m9.figshare.14870136.

10.6084/m9.figshare.14870154 Supplemental Fig. S2: https://doi.org/10.6084/m9.figshare.14870154.

## GRANTS

This research was supported by the JABBS Foundation.

## DISCLOSURES

No conflicts of interest, financial or otherwise, are declared by the authors.

## AUTHOR CONTRIBUTIONS

K.E.J., G.M.B., A.R.B., and S.J.E.L. conceived and designed research; K.E.J., G.M.B., C.B., A.F., and S.J.E.L. performed experiments; K.E.J. and C.B. analyzed data; K.E.J. interpreted results of experiments; K.E.J. prepared figures; K.E.J. and S.J.E.L. drafted manuscript; K.E.J., G.M.B., A.R.B., and S.J.E.L. edited and revised manuscript; K.E.J., G.M.B., C.B., A.F., A.R.B., and S.J.E.L. approved final version of manuscript.
